# Inhibition of MGAT2 modulates fat‐induced gut peptide release and fat intake in normal mice and ameliorates obesity and diabetes in ob/ob mice fed on a high‐fat diet

**DOI:** 10.1002/2211-5463.12778

**Published:** 2020-02-05

**Authors:** Taisuke Mochida, Kazumi Take, Toshiyuki Maki, Masanori Nakakariya, Ryutaro Adachi, Kenjiro Sato, Tomoyuki Kitazaki, Shiro Takekawa

**Affiliations:** ^1^ Cardiovascular and Metabolic Drug Discovery Unit Takeda Pharmaceutical Company Limited Fujisawa Kanagawa Japan; ^2^ Drug Metabolism and Pharmacokinetics Research Laboratories Takeda Pharmaceutical Company Limited Fujisawa Kanagawa Japan; ^3^ Biomolecular Research Laboratories Pharmaceutical Research Division Takeda Pharmaceutical Company Limited Fujisawa Kanagawa Japan

**Keywords:** diabetes, gut hormone, high‐fat diet, MGAT2, Monoacylglycerol O‐acyltransferase 2, obesity

## Abstract

Monoacylglycerol O‐acyltransferase 2 (MGAT2) is one of the key enzymes responsible for triglyceride (TG) re‐synthesis in the small intestine. We have previously demonstrated that pharmacological inhibition of MGAT2 has beneficial effects on obesity and metabolic disorders in mice. Here, we further investigate the effects of MGAT2 inhibition on (a) fat‐induced gut peptide release and fat intake in normal mice and (b) metabolic disorders in high‐fat diet (HFD)‐fed ob/ob mice, a model of severe obesity and type 2 diabetes mellitus, using an orally bioavailable MGAT2 inhibitor Compound B (CpdB). CpdB inhibited elevation of plasma TG in mice challenged with an oil‐supplemented liquid meal. Oil challenge stimulated the secretion of two gut anorectic hormones (peptide tyrosine–tyrosine and glucagon‐like peptide‐1) into the bloodstream, and these responses were augmented in mice pretreated with CpdB. In a two‐choice test using an HFD and a low‐fat diet, CpdB selectively inhibited intake of the HFD in normal mice. Administration of CpdB to HFD‐fed ob/ob mice for 5 weeks suppressed food intake and body weight gain and inhibited elevation of glycated hemoglobin. These results indicate that pharmacological MGAT2 inhibition modulates fat‐induced gut peptide release and fat intake in normal mice and improves obesity and diabetes in HFD‐fed ob/ob mice and thus may have potential for development into a treatment of obesity and its related metabolic diseases.

## Graphical abstract

Monoacylglycerol O‐acyltransferase 2 (MGAT2) is responsible for triglyceride re‐synthesis in the small intestine. Here, we investigate the effects of MGAT2 inhibition on (a) fat‐induced gut peptide release and fat intake in normal mice and (b) metabolic disorders in ob/ob mice fed on a high‐fat diet, a model of severe obesity and type 2 diabetes, using a selective MGAT2 inhibitor.

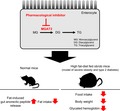

**Abbreviations**
AUC: area under the curve
CpdB: Compound B
GLP‐1: glucagon‐like peptide‐1
HFD: high‐fat diet
MGAT2: monoacylglycerol O‐acyltransferase 2
PYY: peptide tyrosine–tyrosine

Ingested nutrients act on the central nervous system directly as signaling molecules and also by indirectly utilizing signals derived from peripheral tissues, which results in maintenance of whole‐body metabolic homeostasis. One of the indirect mechanisms is so‐called ‘fat sensing’ in the gastrointestinal tract 1, 2, 3, 4, 5, 6, 7, 8. For example, the exposure of receptors to fat—specifically fatty acids in the small intestine—stimulates release of gut peptides that regulate appetite and whole‐body energy balance 3, 4, 5. The ingestion of fat also triggers the production of mediators that play important roles in regulating gastrointestinal motility and the brain reward system for high‐fat food intake by leading activation of vagal afferents 6, 7, 8. A number of reports have suggested the relevance of disrupting these fat‐sensing mechanisms in the pathophysiology of metabolic diseases, such as obesity and type 2 diabetes 1, 2, 3, 4, 5, 6, 7.

Monoacylglycerol O‐acyltransferase 2 (MGAT2) catalyzes the synthesis of diacylglycerol from free fatty acid (FFA) and monoacylglycerol (MG), which are the two major hydrolysis products of dietary fat 9. MGAT2 is highly expressed in the small intestine and is the rate‐limiting enzyme for the triacylglycerol re‐synthesis pathway, the MG pathway, in enterocytes 10, 11. In mice, genetic deletion of MGAT2 alters the spatial distribution of fat absorption in the small intestine and protects against diet‐induced obesity and glucose intolerance in mice 12, 13. Enhanced release of anorectic gut peptides, such as glucagon‐like peptide‐1 (GLP‐1), and altered macronutrient preferences shifted away from fat were also observed in MGAT2 knockout (KO) mice 12, 14. These reports suggest that MGAT2 is a key molecule involved in fat sensing in the gut and support the concept that pharmacological inhibition of MGAT2 could be used as a treatment for obesity and its related metabolic diseases. Several small molecule MGAT2 inhibitors have been identified so far 15. However, a limited number of studies have reported on the efficacy of pharmacological MGAT2 inhibition for metabolic diseases in preclinical studies 16.

We previously reported a selective, potent, and orally bioavailable MGAT2 inhibitor, Compound A (CpdA) 17. CpdA showed anorectic effect in mice fed a high‐fat diet (HFD) and ameliorated hyperglycemia and fatty liver in a severe diabetes mouse model that had been induced by HFD and streptozotocin 18. In the current study, we report a further investigation of the effects of MGAT2 inhibition on (a) fat‐induced gut peptide release and fat intake in normal mice and (b) metabolic disorders in HFD‐fed ob/ob mice—a model of severe obesity and type 2 diabetes mellitus—using another MGAT2 inhibitor, Compound B (CpdB). We found that CpdB showed potent and selective MGAT2 inhibitory activity comparable to CpdA 19. Our data suggest several new beneficial aspects of pharmacological MGAT2 inhibition for improvement of obesity‐related metabolic diseases in mice.

## Materials and methods

### Materials

CpdB, CpdA, and pioglitazone were synthesized at Takeda Pharmaceutical Co., Ltd. (Tokyo, Japan). The *in vitro* inhibitory activities of CpdB and CpdA against MGAT2 and related acyltransferases (DGAT1, DGAT2, and ACAT1) were evaluated as described in our previous report 17 and patent application 19. Liraglutide (Victoza) was purchased from Novo Nordisk Pharma Ltd. (Bagsvaerd, Denmark). All other chemicals were of analytical grade and purchased from Wako Pure Chemicals (Osaka, Japan).

### Animals

Male C57BL/6J mice were purchased from CLEA Japan, Inc. (Tokyo, Japan). Male B6.Cg‐Lepob/J mice (ob/ob mice) were purchased from Charles River Japan, Ltd. (Kanagawa, Japan). Animals had *ad libitum* access to normal chow (CE‐2; CLEA Japan, Inc.) and tap water unless otherwise stated. Animals were housed individually under controlled temperature (20–26 °C), humidity (40–70%), and a 12‐h light/12‐h dark cycle (lights on 7:00–19:00). All animal experiments were conducted in accordance with the protocol reviewed by the institutional animal care and use committee of Takeda Pharmaceutical Company, Ltd.

### Oral meal tolerance test (MTT)

Overnight‐fasted C57BL/6J mice underwent an MTT in the morning. Vehicle, CpdA (3, 10 mg·kg^−1^), or Cpd B (3, 10 mg·kg^−1^) was administered by gavage. The compound was suspended in 0.5% methylcellulose. Six‐and‐a‐half or 16.5 h after gavage, pluronic F‐127 (500 mg·kg^−1^, BASF, Ludwigshafen, Germany) was intraperitoneally injected to inhibit plasma triglyceride (TG) hydrolysis by lipoprotein lipase. Thirty minutes after injection, liquid meal (10 mL·kg^−1^) comprising an admixture of corn oil and Ensure‐H (3 : 17 v/v) (Abbott Japan Co., Ltd., Tokyo, Japan) was orally loaded. Blood samples were collected preload (defined as 0 h) and 2 and 4 h after fat load, and plasma TG levels were measured. The area under the curve (AUC) of chylomicron TG (CM/TG), which is synthesized from dietary fat in the small intestine, was calculated by subtracting the plasma TG levels of the meal‐unloaded group from that of the meal‐loaded group.

### Pharmacokinetics of CpdB in mice

Fifty‐four‐week‐old C57BL/6J mice fed an HFD (45% kcal fat, 4.7 kcal·g^−1^; D12451; Research Diets, Inc., New Brunswick, NJ, USA) were given a single oral administration of CpdB (30 mg·kg^−1^). Blood samples were collected at 0.25, 0.5, 1, 2, 4, 8, and 24 h after the administration. Concentration of plasma CpdB in each sample was measured using liquid chromatography with tandem mass spectrometry as described in our previous report 18.

### Evaluation of the effect on fat‐induced gut peptide release

Overnight‐fasted C57BL/6J mice were divided into groups based on body weight and orally administered vehicle or CpdB (10 mg·kg^−1^). CpdB was suspended in 0.5% methylcellulose. Thirty minutes after administration, fat was loaded via oral gavage of either olive oil (8 mL·kg^−1^) or liquid meal as described above (10 mL·kg^−1^). Blood samples were collected precompound administration (defined as 0 h) and 1, 2, and 3 h after fat loading. Plasma levels of peptide tyrosine–tyrosine (PYY) and GLP‐1 were measured. To confirm the *in vivo* efficacy of CpdB, plasma TG levels were measured 2 h after olive oil loading. Mice orally loaded with water (8 mL·kg^−1^) following vehicle administration were prepared as the control group.

### Food choice test

The choice between HFD (D12451; Research Diets, Inc.) and low‐fat diet (LFD, 10% kcal fat, 3.8 kcal·g^−1^; D124510B; Research Diets, Inc.) was assessed in C57BL/6J mice. Following habituation to normal chow, HFD and LFD were presented in separate containers simultaneously, and overnight food intake of each diet was monitored. The mice were divided into groups based on the data of food intake and body weight. Then, vehicle or CpdB (10 mg·kg^−1^) was orally administered, and the overnight food intake of each diet was monitored. CpdB was suspended in 0.5% methylcellulose. As a positive control, liraglutide (0.04 mg·kg^−1^) dissolved in 10% DMSO/saline was subcutaneously administered.

### HFD‐fed ob/ob mice study

Male ob/ob mice were fed an HFD (D12451; Research Diets, Inc.) from 8 weeks of age to the end of the study. After 2 weeks of HFD feeding, the mice were divided into groups based on their body weight, food intake, glycated hemoglobin (GHb), and plasma biochemical parameters. Vehicle, pioglitazone (3 mg·kg^−1^), or CpdB (30 mg·kg^−1^) was administered orally once daily for 36 days. Compounds were suspended in 0.5% methylcellulose. Body weight and food intake were monitored during the study. On day 34, blood was collected and GHb and plasma biochemical parameters were measured. In addition, body composition was analyzed by EchoMRI‐900 (Hitachi Aloka Medical, Ltd., Tokyo, Japan). On day 36, the mice in the vehicle‐ and CpdB‐treated group were individually placed in the metabolic chamber of an Oxymax system (Columbus Instruments, Columbus, OH, USA). After 3 h of adaptation, oxygen consumption (VO_2_) and carbon dioxide production (VCO_2_) were analyzed for 22 h (from 13:00 to 11:00). During the measurement, dosing of vehicle or CpdB was performed at 18:00. Respiratory quotient (RQ) and energy expenditure (EE) were calculated with the following formulas:

$$\text{RQ}={\text{VCO}}_{2}/{\text{VO}}_{2}$$

$$\text{EE}\left(\text{kcal}/h\right)=\left(3.815+1.232\times \left({\text{VCO}}_{2}/{\text{VO}}_{2}\right)\right)\times {\text{VO}}_{2}$$

### Measurement

Blood samples were collected from the tail or facial vein without anesthesia and centrifuged at 1500 ***g*** for 10 min at 4 °C to isolate the plasma. To prevent degradation of incretin hormone, blood samples were treated with not only heparin/aprotinin but also a dipeptidyl peptidase‐4 (DPP‐4) inhibitor. Plasma alanine aminotransferase (AST) and aspartate aminotransferase (ALT) levels were measured by an Autoanalyzer 7180 (Hitachi High‐Technologies Corporation, Tokyo, Japan). GHb was measured with an automated GHb analyzer (HLC‐723G8; Tosoh, Tokyo, Japan). Plasma insulin was measured with an ELISA kit (Shibayagi Co., Ltd., Gunma, Japan). Plasma levels of total GLP‐1 and total PYY were also measured with an ELISA kit (Wako Pure Chemicals).

### Statistics

Data are expressed as the means and SD values. Statistical differences between two groups were analyzed with Student’s *t*‐test or Aspin–Welch test. Alternatively, statistical significance was first analyzed using Bartlett's test for homogeneity of variances, followed by the one‐tailed William's test for dose‐dependent studies, and Dunnett’s test and Tukey's test for multiple comparisons.

## Results

### Potent and selective inhibitory activity of CpdB for MGAT2 *in vitro*

**Figure 1:**
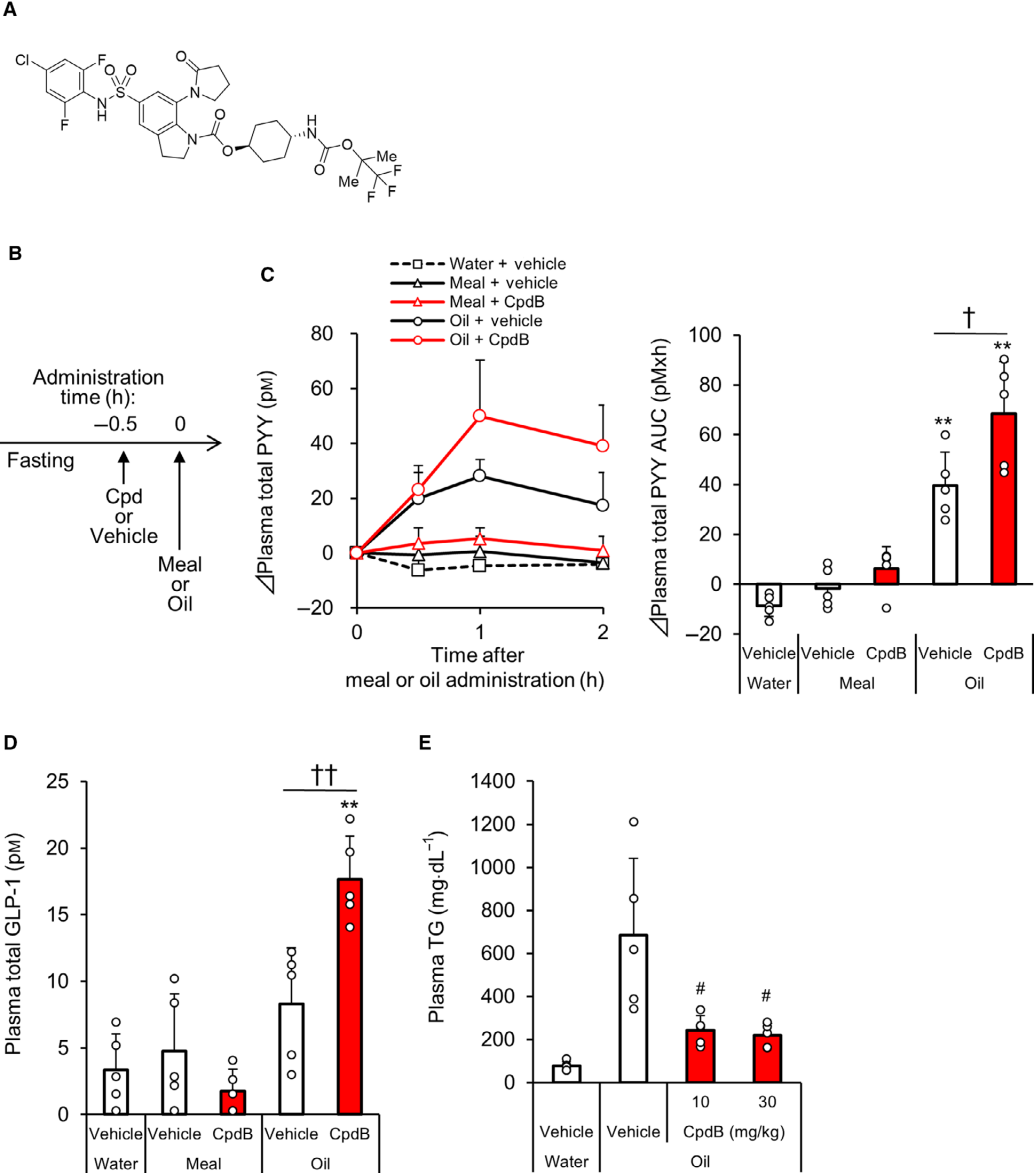
The effect of CpdB on plasma gut peptide levels in meal‐ or oil‐loaded C57BL/6J mice. Fasted C57BL/6J mice were loaded with a liquid meal or oil following administration of CpdB (10 mg·kg^−1^). (A) Chemical structure of CpdB. (B) The brief schematic diagram of the experimental procedure. (C) Changes in plasma total PYY levels for 2 h after meal or oil load (0–2 h) and the AUC. (D) Plasma total GLP‐1 levels at 3 h. (E) Plasma TG levels at 2 h after oil load. **P < 0.01 vs. water‐vehicle by Dunnett's test. ^†^ *P* < 0.05, ^††^ *P* < 0.01 vs. oil‐vehicle by Tukey's test. ^#^ *P* < 0.025 vs. vehicle group by one‐tailed Williams’ test. Data are represented as the mean and SD values (*N* = 5).

As described in our patent application 19, CpdB had a potent inhibitory activity against human and mouse MGAT2 (Table 1 and Fig. 1A). The IC_50_ values for human and mouse MGAT2 were 8.1 and 0.85 nm, respectively. CpdB exhibited greater than 300‐fold selectivity against related acyltransferases (DGAT1, DGAT2, and ACAT1). Those profiles were almost same as a previously reported MGAT2 inhibitor CpdA of which IC50 values for human and mouse MGAT2 were 7.8 and 2.4 nm, respectively 17.

**Table 1:** The *in vitro* inhibitory activities of CpdB for MGAT2 and related acyltransferases.

	MGAT2 human/mouse	DGAT1 human	DGAT2 human	ACAT1 human
IC_50_ (nm)	8.1/0.85	2500	> 30 000	> 30 000

### The inhibitory effect of CpdB on fat‐induced hypertriglyceridemia in C57BL/6J mice

To evaluate the anti‐hypertriglyceridemic effect of CpdB, we investigated changes in plasma TG levels in a mouse MTT model using CpdA as a positive control. CpdB, which had been orally administered 6.5 h before the meal challenge (−6.5 h), inhibited plasma TG elevation and significantly decreased the AUC of plasma CM/TG, and the decreasing effect plateaued at 3 mg·kg^−1^ (Fig. S1A,B). At 10 mg·kg^−1^, CpdB decreased the plasma CM/TG AUC by 37% compared with the vehicle group. A similar level of the decrease was also shown when CpdB was administered at −16.5 h (Fig. S1C,D). The anti‐hypertriglyceridemic effect of CpdB was almost the same as CpdA. CpdA was suggested to achieve MGAT2 inhibition for 24 h by the single dosing in mice, including pharmacokinetics data 18. When CpdB was orally administered at 30 mg·kg^−1^ in mice fed an HFD, the average plasma CpdB levels 24 h after the administration were 0.58 μm (Fig. S2), which was ~ 700 times higher than the IC50 value for mouse MGAT2 enzyme (Table 1). Compared with this, plasma CpdB levels were higher even when administered at 10 mg·kg^−1^ in several experiments in normal mice (data not shown). Based on the anti‐hypertriglyceridemic effect (Fig. S1D) and the pharmacokinetic data, single dosing of CpdB at 10 mg·kg^−1^ was suggested to be able to achieve MGAT2 inhibition for 24 h in mice. Therefore, we selected doses of at least 10 mg·kg^−1^ CpdB for all subsequent experiments.

### An enhancing effect of CpdB on fat‐induced gut peptide release in C57BL/6J mice

To investigate the effect of pharmacological MGAT2 inhibition on gut peptide release, normal C57/BL6J mice were loaded with a liquid meal or oil via oral gavage after administration of CpdB (10 mg·kg^−1^) (Fig. 1B). The oil loading significantly increased plasma levels of total PYY. The increase was significantly enhanced by pre‐administration of CpdB compared with the vehicle (Fig. 1C). In addition, CpdB significantly enhanced oil‐induced plasma total GLP‐1 increase at 3 h after oil load (Fig. 1D). In contrast, increases in plasma levels of total PYY and total GLP‐1 were not detected after liquid meal loading with either the vehicle or CpdB treatments. CpdB potently inhibited the elevation of plasma TG at 2 h after oil load, and the inhibitory effect plateaued at 10 mg·kg^−1^ (Fig. 1E), indicating that enough MGAT2 inhibition was achieved in this experiment model. Based on these results, pharmacological MGAT2 inhibition by CpdB enhanced oil‐induced PYY and GLP‐1 release into the blood stream in normal mice.

### The modulating effect of CpdB on HFD intake in C57BL/6J mice

**Figure 2:**
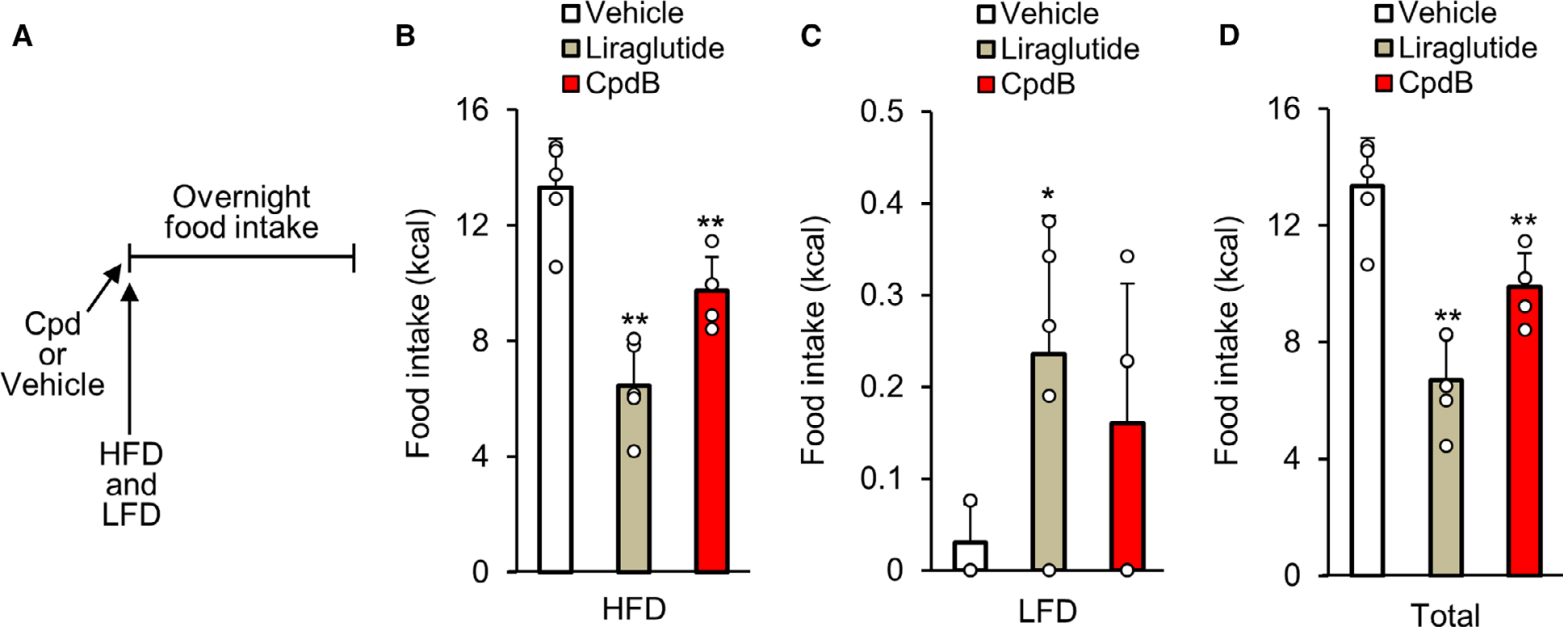
The effect of CpdB on fat intake in a two‐diet‐choice test using C57BL/6J mice. C57BL/6J mice pre‐administered with CpdB (10 mg·kg^−1^) were given an HFD and an LFD simultaneously, and overnight intake of each diet was monitored. (A) The brief schematic diagram of the experimental procedure. Energy intake of (B) HFD, (C) low‐fat diet, and (D) total energy intake. *P < 0.05, **P < 0.01 vs. vehicle by Dunnett's test. Data are represented as the mean and SD values (*N* = 5).

To investigate the effect of CpdB (10 mg·kg^−1^) on intake of HFD, the choice between HFD and LFD was assessed in normal C57BL/6J mice (Fig. 2A). A long‐acting GLP‐1 analogue, liraglutide (0.04 mg·kg^−1^), was used as a positive control since the compound has been reported to preferably reduce the intake of highly palatable diet in rodent 20. Compared with the vehicle treatment, CpdB showed a significant decrease in HFD intake (Fig. 2B) and a tendency to increase that of LFD (Fig. 2C). These changes resulted in significant decrease of total energy intake (Fig. 2D). These results indicated that—although not as clearly as liraglutide—pharmacological MGAT2 inhibition by CpdB selectively inhibited HFD intake and suppressed total energy intake in normal mice.

### Antidiabetic and anti‐obesity effects of CpdB in HFD‐fed ob/ob mice

**Figure 3:**
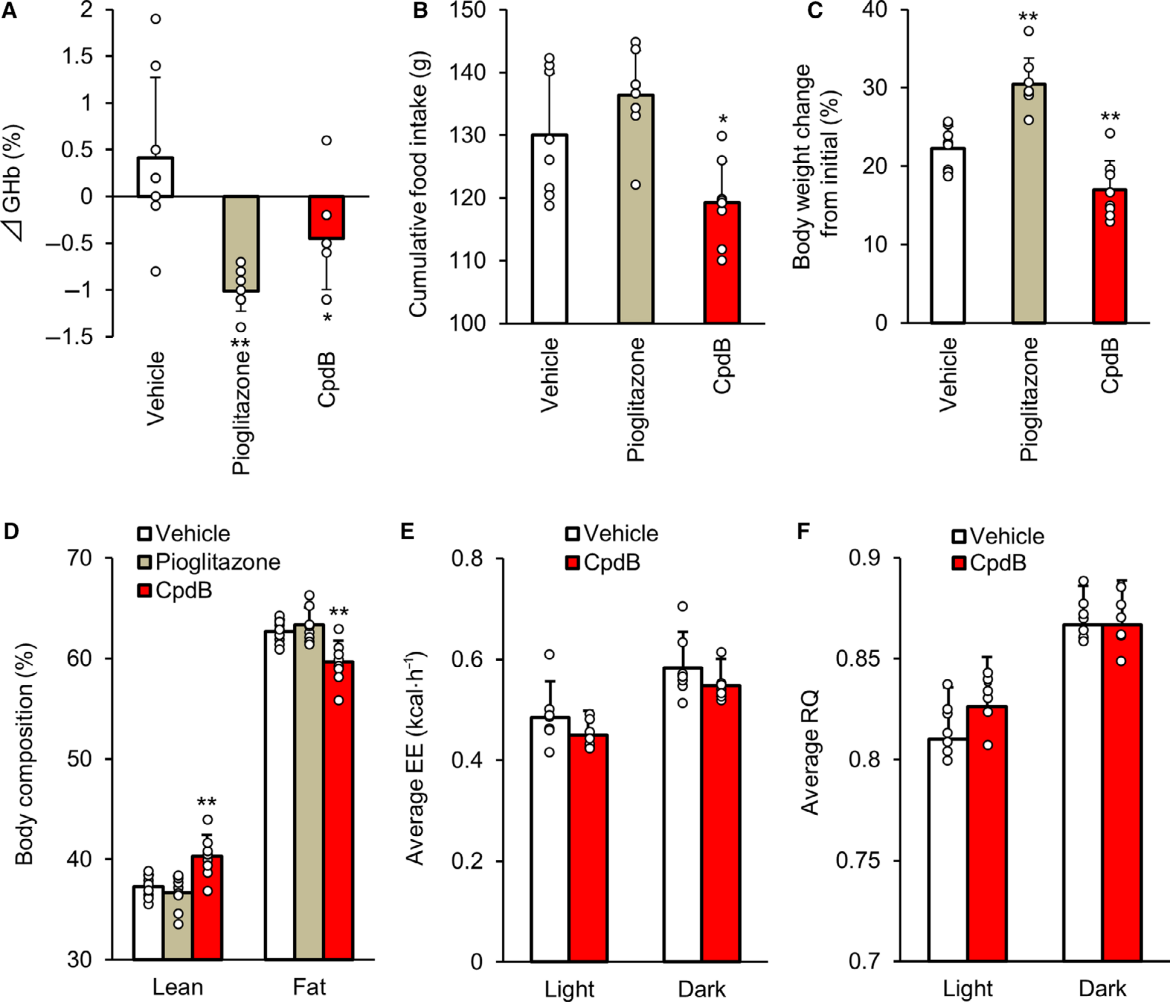
The effect of CpdB on diabetes and obesity in HFD‐fed ob/ob mice. CpdB (30 mg·kg^−1^) was administered orally once daily for 36 days in HFD‐fed ob/ob mice. (A) Changes in GHb levels 34 days after the repeated dosing. (B) Cumulative food intake, (C) % changes in body weight, and (D) lean and fat mass composition measured with EchoMRI. (E) Average EE and (F) RQ during light and dark phase at day 36. *P < 0.05, **P < 0.01 vs. vehicle by Dunnett's test. Data are represented as the mean and SD values (*N* = 7–8).

To investigate the antidiabetic and anti‐obesity effects of pharmacological MGAT2 inhibition, CpdB (30 mg·kg^−1^) was administered orally once daily for 36 days in HFD‐ob/ob mice, which is a model of severe obesity and type 2 diabetes. The insulin sensitizer, pioglitazone, was also evaluated as a positive control since we had previously confirmed that the compound showed clear antidiabetic effect in this animal model (data not shown). Thirty‐four days after the repeated dosing (day 34), CpdB significantly lowered GHb levels compared with the vehicle treatment (Table 2 and Fig. 3A). CpdB decreased cumulative food intake (Fig. 3B), suppressed body weight gain (Fig. 3C), and lowered fat mass composition (Fig. 3D) compared with the vehicle‐treated group. Tendencies to decrease in the plasma levels of AST and ALT, but not insulin, were observed in CpdB‐treated group compared with vehicle‐treated group at day 34 (Table 2). Differences in average EE (Fig. 3E) and RQ (Fig. 3F) were not observed between CpdB‐ and vehicle‐treated mice at day 36. These results indicated that pharmacological MGAT2 inhibition by CpdB suppressed excessive intake of an HFD and improved obesity and diabetes in this animal model.

**Table 2:** Levels of GHb and plasma biochemical parameters in HFD‐fed ob/ob mice treated with the compounds. Data are presented as the mean ± SD values (*n* = 8).

Parameter	Group	Pre	Post
GHb (%)	Vehicle	5.9 ± 0.2	6.4 ± 1.0
	Pioglitazone	5.9 ± 0.3	4.9 ± 0.4**
	CpdB	5.9 ± 0.4	5.4 ± 0.6*
Plasma insulin (ng·mL^−1^)	Vehicle	109 ± 22	89.8 ± 38
	Pioglitazone	103 ± 29	30.8 ± 16**
	CpdB	106 ± 30	109 ± 40
Plasma AST (U·L^−1^)	Vehicle	291 ± 50	611 ± 150
	Pioglitazone	314 ± 61	733 ± 174
	CpdB	333 ± 64	492 ± 122
Plasma ALT (U·L^−1^)	Vehicle	330 ± 26	756 ± 175
	Pioglitazone	347 ± 53	803 ± 132
	CpdB	378 ± 76	632 ± 144

* *P* < 0.05

** *P* < 0.01 vs. Vehicle by Dunnett's test.

## Discussion

The function of the MGAT2 gene is not only related to the assimilation of dietary fat in the small intestine but also regulation of whole‐body homeostasis, such as body weight and glucose homeostasis 15. In this study, we revealed that pharmacological MGAT2 inhibition by CpdB modulated the release of anorectic gut peptides and intake of an HFD in normal mice and showed a beneficial effect on improvement of obesity and diabetes in HFD‐ob/ob mice. Based on the anti‐hypertriglyceridemic effect of CpdB in mouse MTT (Fig. S1B,D) and the pharmacokinetic data in mice as representatively shown in Fig. S1, we consider that once‐daily dosing of CpdB would achieve durable MGAT2 inhibition in our experiments.

Throughout the gastrointestinal tract, specialized endothelial cells, called enteroendocrine cells, sense the luminal content, such as nutrients, and consequently release peptides, such as GLP‐1 and PYY. In response to ingested fat, GLP‐1 and PYY are secreted from L cells in the distal small intestine and the colon 21. These two gut peptides are well known as satiety hormones, and they work in concert to regulate appetite. Postprandial elevation of plasma GLP‐1 levels was observed in MGAT2 KO mice chronically fed a 60% fat diet 12. As for pharmacological MGAT2 inhibition by a small molecule compound, JTP‐103237 was reported to enhance the increase of plasma levels of PYY but not GLP‐1 after lipid loading in rats fed a 35% fat diet 16. In our previous report using CpdA 18, the influence on gut peptide secretion *in vivo* was not investigated. In the present study, we showed that pharmacological MGAT2 inhibition by CpdB was able to enhance the increase of plasma levels of both PYY and GLP‐1 simultaneously in normal mice‐loaded fat (Fig. 1C,D). MGAT2 KO mice are reported to have delayed absorption of dietary fat, such as FFA and MG, from the proximal to distal intestine without fecal lipid excretion 12, 13. In addition, several reports have shown that MG increased GLP‐1 secretion in the GLUTag enteroendocrine cell line 18, 22. It has also been suggested that intestinal accumulation of MG would stimulate GPR119 on L cells resulting in gut hormone releases 23. Therefore, CpdB may delay fat absorption with more FFA and MG reaching the distal intestine and consequently enhance L cell‐derived secretion of GLP‐1 and PYY into the bloodstream. Evaluation in mice lacking receptors, such as GPR40, GPR119, and GPR120 that are involved in gastrointestinal sensing of dietary fat, might offer further information on the mechanism underlying CpdB‐induced gut peptide release. One of our future interests is whether the benefit of enhanced GLP‐1 secretion by CpdB could be extended by combination with a DPP‐4 inhibitor, which is clinically used to enhance incretin action.

An important feature of obesity is deficient dopamine transmission in response to food consumption in the brain reward system, which is hypothesized to play a key role in shifting dietary preferences away from healthier low‐fat food toward more palatable high‐fat food to aggravate obesity 24, 25. An MGAT2 deficiency is reported to shift food preference away from fat and toward carbohydrates when mice are given a food choice, and the shift is not a simple alteration to avoid energy‐dense diets 14. In this study, we showed, for the first time to our knowledge, that a pharmacological MGAT2 inhibitor selectively inhibited intake of an HFD in the mouse food choice test (Fig. 2B–D). A previously reported MGAT2 inhibitor CpdA did not show acute anorectic effect in MGAT2 KO mice 18, and CpdB showed highly selective inhibitory activity for MGAT2 *in vitro* similar to CpdA. Therefore, the inhibitory effect of CpdB on HFD intake seems to be dependent on MGAT2 inhibition. We also considered that malabsorption of dietary fat would not be involved in the result since previous reports showed neither MGAT2 deficiency nor pharmacological MGAT2 inhibition increased fecal fat excretion 12, 18. Several rodent studies highlight the critical role for gut–brain communication in regulating preference for high‐fat palatable food. For example, weight gain disrupts intestinal production of the bioactive lipid amide, oleoylethanolamine (OEA), which serves as a satiety signal for dietary fat. Recovery of intestinal OEA signaling can attenuate deficits in striatal dopaminergic transmission 25. It has also been reported that dietary fatty acids induce cerebral dopamine release, while 2‐oleoyl glycerol does not 26. Apart from this, a long‐acting GLP‐1 analogue, liraglutide, has been reported to predominantly reduce the intake of highly palatable diet in rats 20, implying that the regulation of preference for high‐fat palatable food is controlled by GLP‐1 signaling. To understand the mechanism by which CpdB modulates fat intake, it might be useful to investigate whether CpdB increases the intestinal production of OEA and/or enhances GLP‐1 secretion enough to affect the brain reward system. Of note, longer‐term studies will lead to further understanding of the results in this study, and we cannot completely exclude the influence of nutrients other than the fat and energy density of the diet.

Ob/ob mice exhibit a mutation in the leptin gene that typically results in severe obesity with hyperphagia and hyperglycemia with insulin resistance. These mice are widely used in nutritional and pharmacological studies aimed at the treatment of obesity and type 2 diabetes mellitus 27. Of note, ob/ob mice also show an increased preference for HFD compared with normal mice 28, and the obese phenotype is reported to be further exacerbated when they are maintained on a HFD 29. In the current study, repeated gavage with CpdB suppressed body weight gain and completely inhibited elevation of GHb in HFD‐ob/ob mice (Fig. 3A,C). To our knowledge, this is the first report showing the beneficial effect of an MGAT2 inhibitor on improvement of severe obesity and diabetes in this mouse model. We considered that these effects were exerted mainly via suppression of overeating of the palatable high‐energy diet (Fig. 3B). After a 5‐week repeated gavage, CpdB did not lower the levels of nonfasting plasma insulin levels compared with vehicle treatment (Table 2). In contrast, the insulin sensitizer, pioglitazone, clearly lowered it. In addition, no clear effects on EE and RQ were shown by CpdB in both the light and dark phases (Fig. 3E,F). These results support the hypothesis that reduction of food intake, rather than improvement of insulin resistance and enhancement of fat oxidation in peripheral tissues, contributes to the anti‐obesity and anti‐hyperglycemic effect of CpdB in HFD‐ob/ob mice. Since MGAT2 KO mice maintained on an HFD were reported to show higher oxygen consumption in the dark phase than WT mice 12, it was unexpected that no enhancement of EE was shown by repeated CpdB gavage. Although the details remain unclear, decreased EE by a mutation in the leptin gene 30 and a more severe induction of the obese phenotype through exposure to an HFD 29 might be relevant to the result. Regarding methodology, we might be able to optimize the habituation period to the Oxymax system, but the animals were habituated for 3 h to reduce the influence of stress in this study. Compared with the vehicle treatment, CpdB tended to lower the plasma levels of liver enzymes, such as AST and ALT (Table 2). The tendency seems to be derived mainly from suppression of body weight gain and perhaps partially from a direct effect on the liver, although very low expression levels of MGAT2 in mouse liver have been reported 9, 31. This consideration would be relevant to the potential of an MGAT2 inhibitor for the treatment of nonalcoholic steatohepatitis through improvement of whole‐body metabolism, such as weight loss 15. We have not confirmed whether CpdB can affect the release of anorectic gut peptides in HFD‐ob/ob mice as was shown in normal C57BL/6J mice. Further studies would be required to deeply investigate regarding contribution factors to the efficacy of CpdB in HFD‐ob/ob mice.

An intervention that enables effective weight loss in obese individuals is Roux‐en‐Y gastric bypass (RYGB) surgery, which is the most common type of bariatric surgery 32. It is generally accepted that the surgery results in not only weight loss but also remission of the disease shortly after surgery in obese and type 2 diabetes mellitus patients 33. Exaggerated secretion of gut peptides, such as GLP‐1 and PYY, following meal ingestion 34, reduced appetite for dietary lipids, and decreased sensitivity to the hedonic properties of palatable food 35, 36 were observed in RYGB surgery‐treated patients. These changes have also been reported in rodent models 37 and are considered to contribute to the beneficial effects of RYGB surgery; however, the precise mechanism remains unknown. In the current study, we revealed that CpdB modulated gut peptide release and HFD intake vaguely similar to the RYGB surgery. Whether mechanisms underlying the beneficial effects of RYGB surgery overlap with that of pharmacological MGAT2 inhibition remains to be seen, and further investigation is required.

Collectively, we have demonstrated that pharmacological MGAT2 inhibition modulates fat‐induced gut peptide release and HFD intake, presumably by utilizing fat‐sensing mechanisms in the small intestine. To analyze the mechanism by which CpdB predominantly inhibits intake of an HFD, mice deficient for gut peptide receptors or G protein‐coupled receptors relevant to gastrointestinal sensing of dietary fat might be useful. The obesity pandemic continues to grow, and the ready accessibility of palatable energy‐dense food is considered to be a significant driving force 38. Taken together, newly identified insights into the pharmacological effects of the MGAT2 inhibitor in this study further support the concept that pharmacological MGAT2 inhibition could be used as a treatment for obesity and its related metabolic diseases.

## Conflicts of interest

All authors are employees of Takeda Pharmaceutical Company Limited. The authors are named on the patent application for CpdB.

## Author contributions

TaM involved in the conceptualization of the article; TaM, KT, and ToM performed the methodology of the study; TaM, MN, and RA investigated the article; TaM wrote the original draft of the manuscript; KS and ST wrote, reviewed, and edited the article; ToK and ST supervised the article.

## Supporting information


**Fig. S1.** The effect of CpdB on hypertriglyceridemia during an oral meal tolerance test in mice. Fasted C57BL/6J mice were given a liquid meal orally with intraperitoneal injection of pluronic F‐127 (LPL inhibitor) to inhibit plasma TG lipolysis. (A) The brief schematic diagram of the experimental procedure in (B). (B) Changes in plasma chylomicron TG (CM/TG) levels and the AUC during 4 h after the meal challenge (0–4 h) when CpdB (3 and 10 mg/kg) was administered orally at −6.5 h. CpdA (3 and 10 mg·kg^−1^) was also evaluated as a positive control. (C) The brief schematic diagram of the experimental procedure in (D). (D) Changes in plasma CM/TG levels (0–4 h) and the AUC when CpdA (10 mg·kg^−1^) or CpdB (10 mg·kg^−1^) was administered orally at −16.5 h. #*P* < 0.025 vs. vehicle group by one‐tailed Williams’ test. **P < 0.01 vs. vehicle by Dunnett's test. Data are represented as the mean and SD values (*N* = 6).
**Fig. S2.** Pharmacokinetics of CpdB in mice. Plasma concentrations at 0.25, 0.5, 1, 2, 4, 8, and 24 h after a single oral administration of CpdB (30 mg·kg^−1^). Data are represented as the mean and SD values (*N *= 3).

**Data S1** **. **Data file represents the values and standard deviations of each figure.

## Data Availability

The datasets generated during and/or analyzed during the current study are available from the corresponding author upon reasonable request.
