# Supplementary material for: Inhibition of MGAT2 modulates fat‐induced gut peptide release and fat intake in normal mice and ameliorates obesity and diabetes in ob/ob mice fed on a high‐fat diet
Source: FEBS Open Bio. 2020 Feb 5;10(3):316–26. doi: 10.1002/2211-5463.12778 (PMC7050258; doi:10.1002/2211-5463.12778)
Supplement: Supplementary file 1 — Fig. S1. The effect of CpdB on hypertriglyceridemia during an oral meal tolerance test in mice. Fasted C57BL/6J mice were given a liquid meal orally with intraperitoneal injection of pluronic F‐127 (LPL inhibitor) to inhibit plasma TG lipolysis. (A) The brief schematic diagram of the experimental procedure in (B). (B) Changes in plasma chylomicron TG (CM/TG) levels and the AUC during 4 h after the meal challenge (0–4 h) when CpdB (3 and 10 mg/kg) was administered orally at −6.5 h. CpdA (3 and 10 mg·kg−1) was also evaluated as a positive control. (C) The brief schematic diagram of the experimental procedure in (D). (D) Changes in plasma CM/TG levels (0–4 h) and the AUC when CpdA (10 mg·kg−1) or CpdB (10 mg·kg−1) was administered orally at −16.5 h. #P < 0.025 vs. vehicle group by one‐tailed Williams’ test. **P < 0.01 vs. vehicle by Dunnett's test. Data are represented as the mean and SD values (N = 6). Fig. S2. Pharmacokinetics of CpdB in mice. Plasma concentrations at 0.25, 0.5, 1, 2, 4, 8, and 24 h after a single oral administration of CpdB (30 mg·kg−1). Data are represented as the mean and SD values (N = 3). [file FEB4-10-316-s001.docx]

**Supplementary information**

**
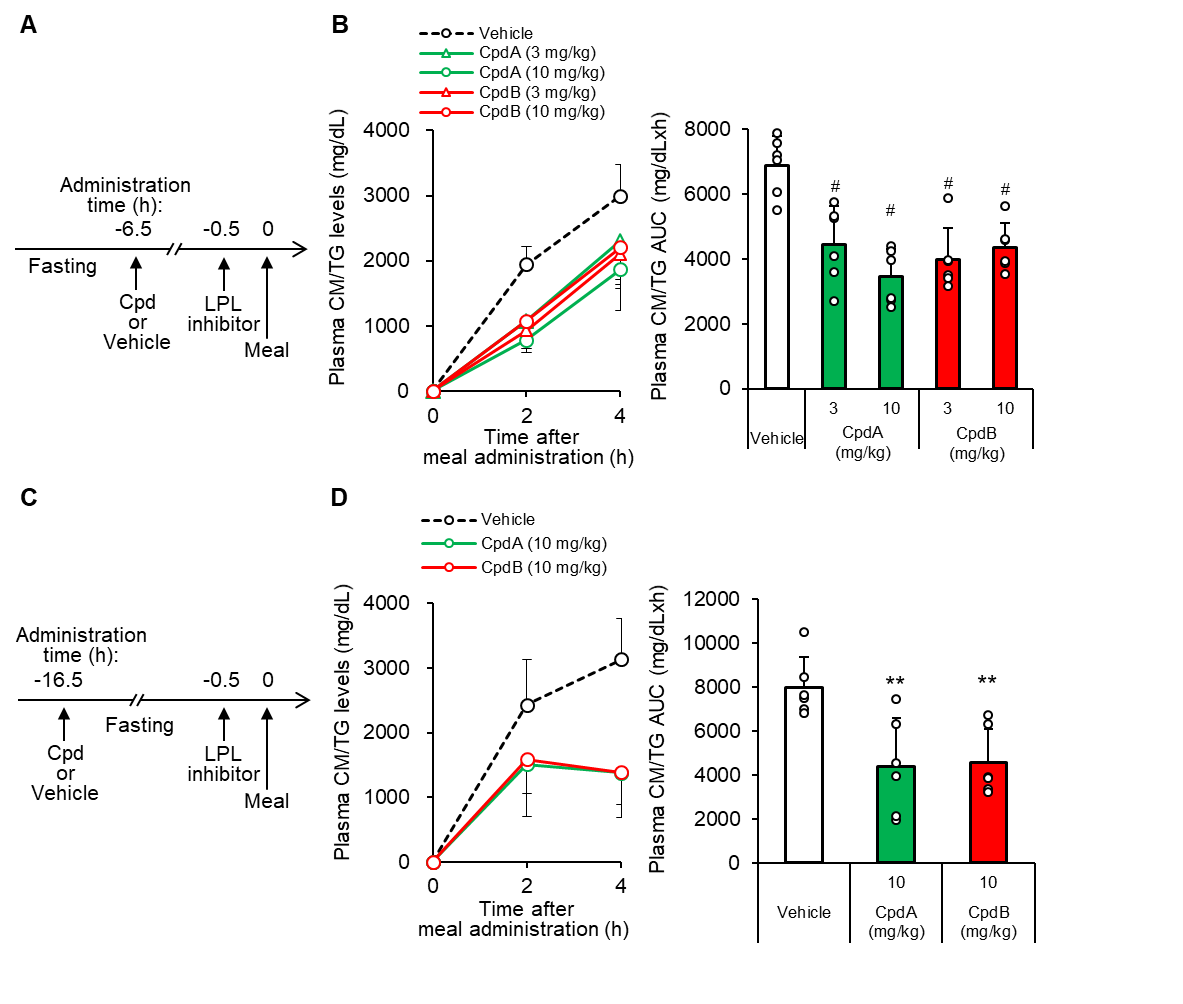
**

**Supplementary figure 1. The effect of CpdB on hypertriglyceridemia during an oral meal tolerance test in mice.** Fasted C57BL/6J mice were given a liquid meal orally with intraperitoneal injection of pluronic F-127 (LPL inhibitor) to inhibit plasma TG lipolysis. (A) The brief schematic diagram of the experimental procedure in (B). (B) Changes in plasma chylomicron TG (CM/TG) levels and the AUC during 4 h after the meal challenge (0-4 h) when CpdB (3 and 10 mg/kg) was administered orally at -6.5 h. CpdA (3 and 10 mg/kg) was also evaluated as a positive control. (C) The brief schematic diagram of the experimental procedure in (D). (D) Changes in plasma CM/TG levels (0-4 h) and the AUC when CpdA (10 mg/kg) or CpdB (10 mg/kg) was administered orally at -16.5 h. #*p* < 0.025 vs. vehicle group by one-tailed Williams’ test. ***p* < 0.01 vs. vehicle by Dunnett's test. Data are represented as the mean and SD values (N=6).


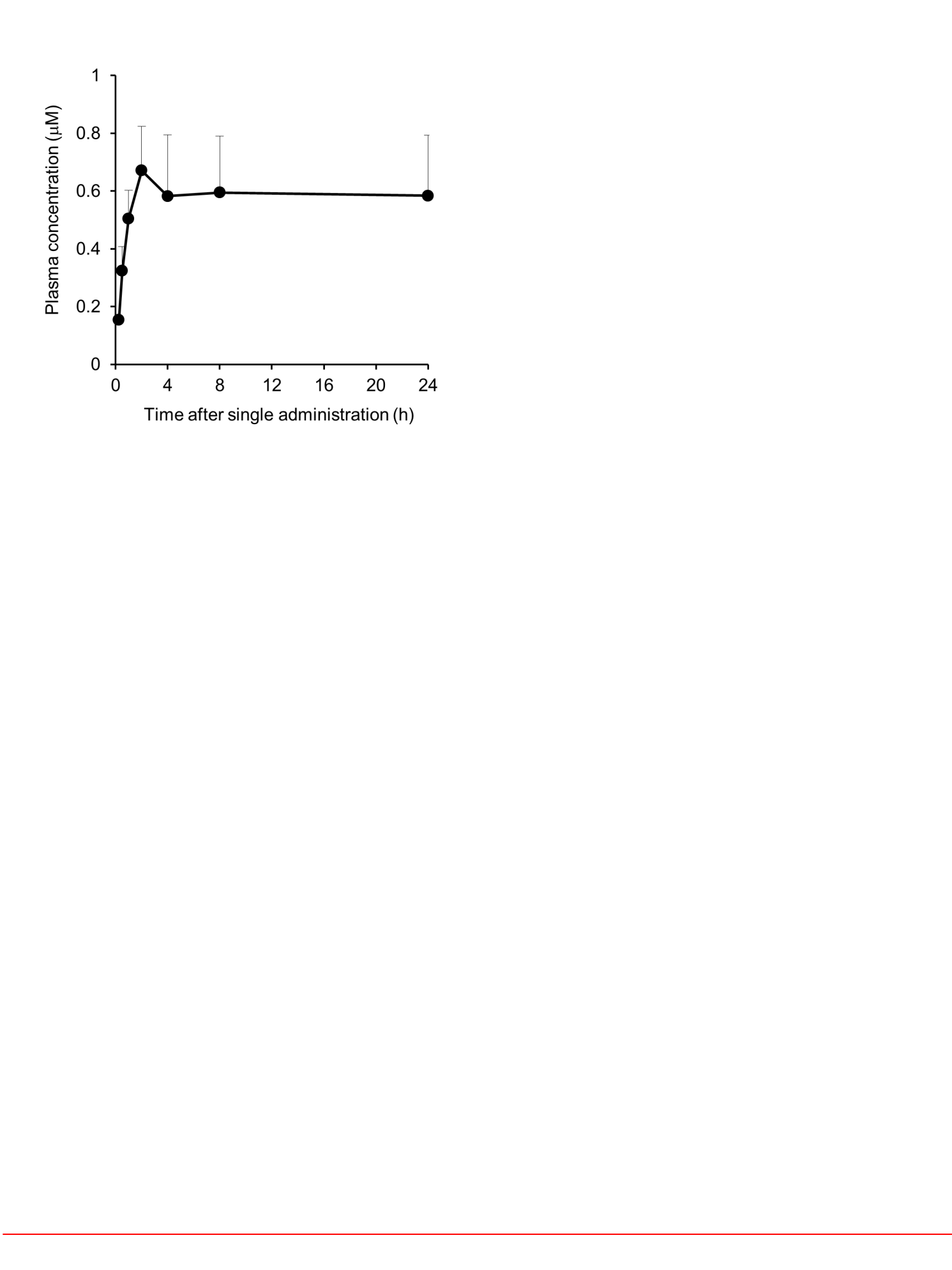


**Supplementary figure 2. Pharmacokinetics of CpdB in mice.** Plasma concentrations at 0.25, 0.5, 1, 2, 4, 8, and 24 h after a single oral administration of CpdB (30 mg/kg). Data are represented as the mean and SD values (N=3).
